# Characteristics and timeliness of intervention in 47 school-based enterovirus outbreaks in Zhejiang Province, China

**DOI:** 10.3389/fpubh.2025.1559637

**Published:** 2025-04-03

**Authors:** Yijuan Chen, Wanwan Sun, Xiaobin Ren, Xiaomin Gu, Kai Song, Pingping Wang, Yanli Cao, Jianmin Jiang, Ziping Miao

**Affiliations:** ^1^Department of Communicable Disease Control and Prevention, Zhejiang Provincial Center for Disease Control and Prevention, Hangzhou, China; ^2^Department of Communicable Disease Control and Prevention, Hangzhou Center for Disease Control and Prevention, Hangzhou, China; ^3^Department of Communicable Disease Control and Prevention, Ningbo Center for Disease Control and Prevention, Ningbo, China; ^4^Pujiang County Center for Disease Control and Prevention, Jinhua, China; ^5^Zhejiang Key Lab of Vaccine, Infectious Disease Prevention and Control, Hangzhou, China

**Keywords:** HFMD, herpangina, outbreaks, timeliness, characteristics

## Abstract

**Background:**

Hand, foot, and mouth disease (HFMD) and herpangina (HA) are common infectious diseases in children that can frequently lead to outbreaks. Analyzing the characteristics of school-based outbreaks and evaluating the timeliness of prevention and control measures can provide valuable insight for early identification, timely warnings, and the development of prevention strategies.

**Methods:**

This study collected data on HFMD and HA outbreaks in Zhejiang Province from 2021 to 2023, providing a comprehensive analysis of the pathogen spectrum, epidemiology, and clinical characteristics of each outbreak. A generalized linear model with a Poisson distribution was used to assess the impact of various intervention timings on the effectiveness of disease control.

**Results:**

Between 2021 and 2023, a total of 47 HFMD and HA outbreaks were reported in Zhejiang Province, spanning eight cities and 28 counties. Specifically, six HA outbreaks were reported in one city and three counties, 31 HFMD outbreaks occurred across eight cities and 22 counties, and ten mixed HFMD-HA outbreaks were identified in four cities and six counties. Herpangina outbreaks were confined to 2021, whereas HFMD outbreaks occurred annually. Nearly all outbreaks (93.62%) occurred in educational or childcare settings. The predominant strain of HFMD shifted from CVA16 in 2021 and 2022 to CVA6 in 2023, while CVA4 was primarily associated with HA. Seasonally, HA outbreaks peaked between April and May, whereas HFMD outbreaks transitioned from a broader March–October season to a narrower May–June period. Clinically, HA cases exhibited a higher proportion of fever, oral herpes, and sore throat compared to HFMD cases (*p* < 0.05). Outbreak duration was positively correlated with the number of cases, with each additional day of outbreak duration leading to a 6.6% increase in case numbers. Similarly, delays in implementing class suspensions were associated with larger outbreaks, with each day’s delay resulting in a 5.0% increase in cases numbers.

**Conclusion:**

Different enteroviruses are responsible for HFMD and HA outbreaks, with childcare facilities frequently acting as key hotspots. Timely case isolation and the implementation of effective management strategies are crucial for controlling the transmission dynamics of these diseases.

## Introduction

Hand, foot, and mouth disease (HFMD) and herpangina (HA) are common childhood infectious diseases caused by human enteroviruses, primarily affecting children under 5 years old, particularly in outbreak-prone settings such as schools and childcare facilities. Since 1997, the Asia-Pacific region has experienced multiple HFMD/HA epidemics, posing significant challenges to local healthcare and public health systems (1–3). Following a severe HFMD outbreak in Fuyang in 2008, which was associated with a high mortality rate, China classified HFMD as a Class C notifiable infectious disease. Subsequently, the number of reported HFMD cases consistently ranked among the top three legally reported infectious diseases nationwide each year.

The incidence rate of HFMD increases every other year, and exhibits obvious seasonal characteristics, with cases peaking during the spring and summer months (April to July) and showing a secondary peak in the autumn (September to November). This periodic fluctuation is influenced by a combination of factors, including meteorological factors, the cyclical activity of virus strains, and population immunity levels (4, 5). Significant regional differences in HFMD incidences are observed across China (6), with particularly high rates in the Yangtze River Delta region (6, 7), especially in Zhejiang Province. Herpangina also demonstrates seasonal trends, peaking primarily in the spring and summer (8), but it is not classified as a notifiable disease. As a result, research on HA has been limited and relies primarily on localized surveys. Studies from Beijing, Jiangsu, and Zhejiang have reported notable incidences of HA (9–11); however, a nationwide monitoring system for HA is currently lacking.

The incubation period for HFMD typically ranges from 2 to 10 days, with an average of 3–5 days. Patients often present with mild symptoms, including rashes on the hands, feet, and buttocks, as well as herpetic sores around the mouth, on the mucous membranes of the cheeks, and under the tongue (12). These symptoms are frequently accompanied by mild fever (below 38°C). Severe complications, such as aseptic meningitis and neurogenic pulmonary edema, can occur, often associated with Enterovirus 71 (EV71) infection (13, 14). Herpangina, which shares a similar incubation period with HFMD, typically presents in children with sudden pharyngeal pain and fever, along with multiple herpetic lesions or ulcers in the posterior pharyngeal region, including the pharyngeal palatine arch, soft palate, uvula, and tonsils (8). While most HA cases are self-limiting, a small proportion may progress to severe conditions, such as high fever with seizures or encephalitis (15). It is worth noting that early symptoms of HFMD, such as oral herpetic sores, can complicate the clinical differentiation between HFMD and HA.

Enteroviruses (EVs) responsible for HFMD and HA belong to the *Picornaviridae family*, genus *Enterovirus*. The International Committee on Taxonomy of Viruses (ICTV) classifies human-infectious EVs into four primary species: Enterovirus A, B, C and D. Enterovirus A (EV-A) includes major HFMD pathogens such as EV71 and coxsackieviruses (CV) A2, A4, A6, A10, and A16, while Enterovirus B (EV-B) encompasses coxsackieviruses B1-B6 and A9. Globally, more than 100 types of enteroviruses have been identified (16). Before 2013, HFMD cases in China were predominantly caused by EV71 and CVA16; however, in recent years, other enteroviruses, such as CVA6 and CVA10, have emerged as significant pathogens, with multiple strains alternating in dominance (6, 9, 17). The predominant pathogens of HA are similar to those of HFMD, with recent dominant strains including CVA2, A4, A6, A10, and A16 (8, 11, 18). Although the introduction of the EV71 vaccine in China in 2016 significantly reduced the incidence of EV71-related diseases (19–21), it does not provide cross-protection against other prevalent pathogens, such as CVA6 and CVA16, which continue to pose challenges for HFMD and HA prevention and control (22). Therefore, ongoing virological research is crucial to improving the diagnosis, treatment, and prevention of these diseases and to supporting the development of polyvalent vaccines (22–24).

In general, both HFMD and HA are enterovirus-induced infectious diseases with similar onset mechanisms, clinical features, and prognoses; however research on HA has been significantly lacking compared to HFMD, particularly in the areas of disease prevention, control, and studies on its association with HFMD. After the inclusion of HFMD in China’s legal infectious disease management system, a series of prevention and control guidelines and work standards were formulated. These include the *Guidelines for Prevention and Control of Hand, Foot and Mouth Disease* (2009 Edition) (25) and the *Guidelines for Handling Cluster and Outbreak Epidemics of Hand, Foot and Mouth Disease* (2012 Edition) (26). These documents provide specific requirements for reporting, investigating, and handling HFMD outbreaks. Zhejiang Province has referred to these guidelines when managing outbreaks of other enterovirus infections, such as HA.

This study aims to provide a comprehensive analysis of the epidemiological characteristics of recent HFMD and HA outbreaks in schools across Zhejiang Province, focusing on pathogen spectra, clinical manifestations, and outbreak dynamics, while exploring the relationship between the timing of interventions, such as school closures, and the scale of outbreaks, which to some extent reflects the timeliness of current prevention and control measures. By systematically comparing these two diseases, this study seeks to improve early detection, timely intervention, and vaccine development to help prevent associated epidemics.

## Materials and methods

### Data sources

The data on HFMD and HA outbreaks from January 1, 2021, to December 31, 2023, were obtained from the “Emergency Public Health Event Reporting Management System” (EPHERMS) and the “China Disease Surveillance Information Reporting Management System” (CDSIRMS).

### Definitions

An outbreak was defined as the occurrence of ≥10 HFMD cases within a week at the same childcare institution or school, or ≥ 5 cases within the same village or community (26). A major outbreak was defined as one with more than 30 reported cases in this study. The criteria for HA outbreaks were consistent with those for HFMD. Criteria for school or childcare closure (25): If severe cases or deaths occur, or if a single class reports ≥2 cases within a week, the affected class is recommended to suspend activities for 10 days. Furthermore, if ≥10 cases are reported within a week, or if three or more classes each report ≥2 cases, the entire school or childcare institution may be recommended to close for 10 days, based on a comprehensive risk assessment.

### Investigation and intervention methods

For HFMD and HA outbreak events, county- or district-level Centers for Disease Control and Prevention (CDCs) conduct epidemiological investigations within 24 h of receiving a report (26). These investigations include on-site visits to identify the index case or initial cases, active case searching, etiological testing, and providing guidance to affected institutions on implementing control measures. Simultaneously, risk assessments are conducted, and recommendations for class or facility closures are made when necessary. Additional measures implemented by local CDCs or recommended to collective institutions include: (1) Conducting further case searches to identify additional infections; (2) Performing environmental disinfection measures, particularly targeting items used by infected children (e.g., toys, utensils, tableware) and surfaces in activity areas; (3) Providing guidance to institutions on the standardized implementation of morning and midday health screenings; (4) Strengthening the documentation and monitoring of absences due to illness; (5) Distributing health education materials to improve public awareness; (6) Advising parents to closely monitor the health of children during school or class closures.

### Classification standards

Outbreaks in which all cases exhibit only oral herpes or ulcers, without rashes on other parts of the body, were classified as HA outbreaks. Outbreaks in which all cases exhibited HFMD-associated rashes (not limited to the oral area), or where clinical symptoms were incompletely described but were uniformly designated as HFMD by the investigators, were classified as HFMD outbreaks. Outbreaks explicitly categorized as involving both HFMD and HA cases in investigation reports were classified as mixed HFMD(HA) outbreaks.

### Inclusion and exclusion criteria

Data on HFMD and HA outbreak events were compiled by professionals. Outbreaks that did not meet the case and outbreak criteria were excluded. A total of 47 outbreaks that met the criteria were included in the basic characteristic analysis. For the clinical analysis, 33 outbreaks with detailed clinical investigation data were included, while 42 outbreaks with sufficient virological test results were included in the virological analysis.

### Specimen collection and testing

For each outbreak, at least five throat swabs or fecal specimens were collected from affected cases. Real-time fluorescence PCR test kits were used to detect the presence of enterovirus nucleic acids.

### Statistical analysis

Data were organized and summarized using Excel 2021, while R software (version 4.3.1) was used for graphing and statistical analysis. Categorical variables were described using frequencies and proportions. Groups comparisons were performed using the Chi-square test or Fisher’s exact test, with pairwise comparisons adjusted by the Bonferroni correction. A generalized linear model (GLM) with a Poisson distribution was applied to explore the relationship between the duration of individual outbreaks and the number of cases. The core formula used was: model_formula = “case ~ duration + start_to_stop + duration*start_to_stop + C(group).” Here, “duration” represents the time from the onset of the first case to the onset of the last case within each outbreak, “start_to_stop” represents the time from the first case onset to the implementation of school closure for each outbreak, and “group” refers to the three previously defined outbreak categories. The significance level was set at *α* = 0.05.

### Ethical statement

The investigation was conducted in response to the public health emergency and received approval from the Ethics Committee of Zhejiang Provincial CDC (approval number: 2016020). Research involving human participants was conducted in accordance with the Declaration of Helsinki.

## Results

### Overview

**Figure 1:**
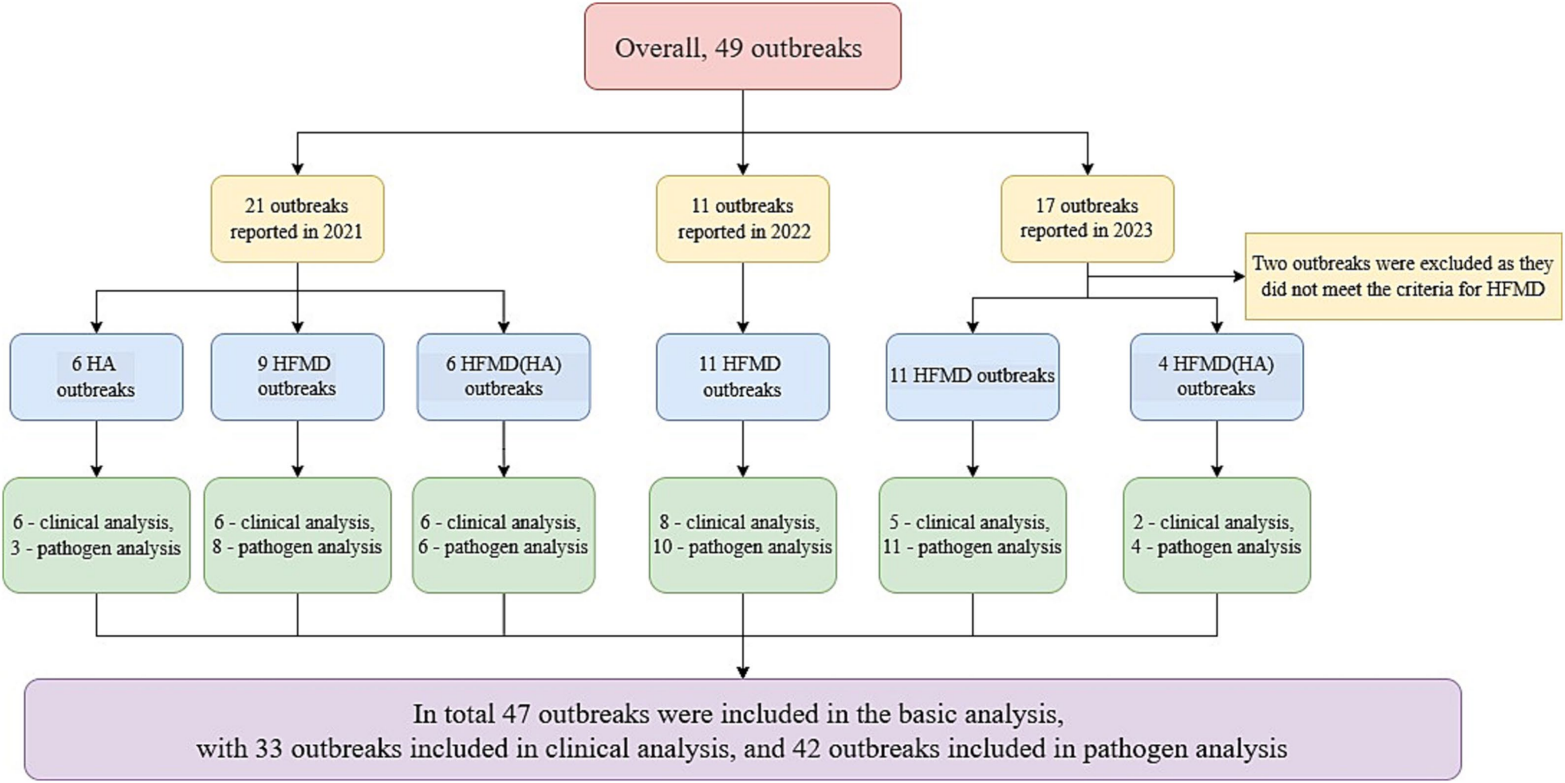
Flowchart of HFMD/HA outbreaks.

Between 2021 and 2023, a total of 49 outbreaks related to HFMD or HA, including four major outbreaks, were reported in Zhejiang Province. In 2021, 21 outbreaks were recorded, including six HA outbreaks, nine HFMD outbreaks, and six HFMD(HA) outbreaks, affecting 357 individuals, with one classified as a major outbreak. In 2022, 11 HFMD outbreaks were reported, involving 217 individuals, including one major outbreak. In 2023, 17 outbreaks were initially reported; however two were later excluded due to misdiagnosis, leaving 15 confirmed outbreaks - 11 HFMD outbreaks and four HFMD(HA) outbreaks - affecting 266 individuals, with two classified as major outbreaks (Figure 1). Among the 47 confirmed outbreaks, all were reported in educational or childcare settings, including 43 in kindergartens, three in elementary schools, and one in a Nursery.

### Geographical distribution

**Figure 2:**
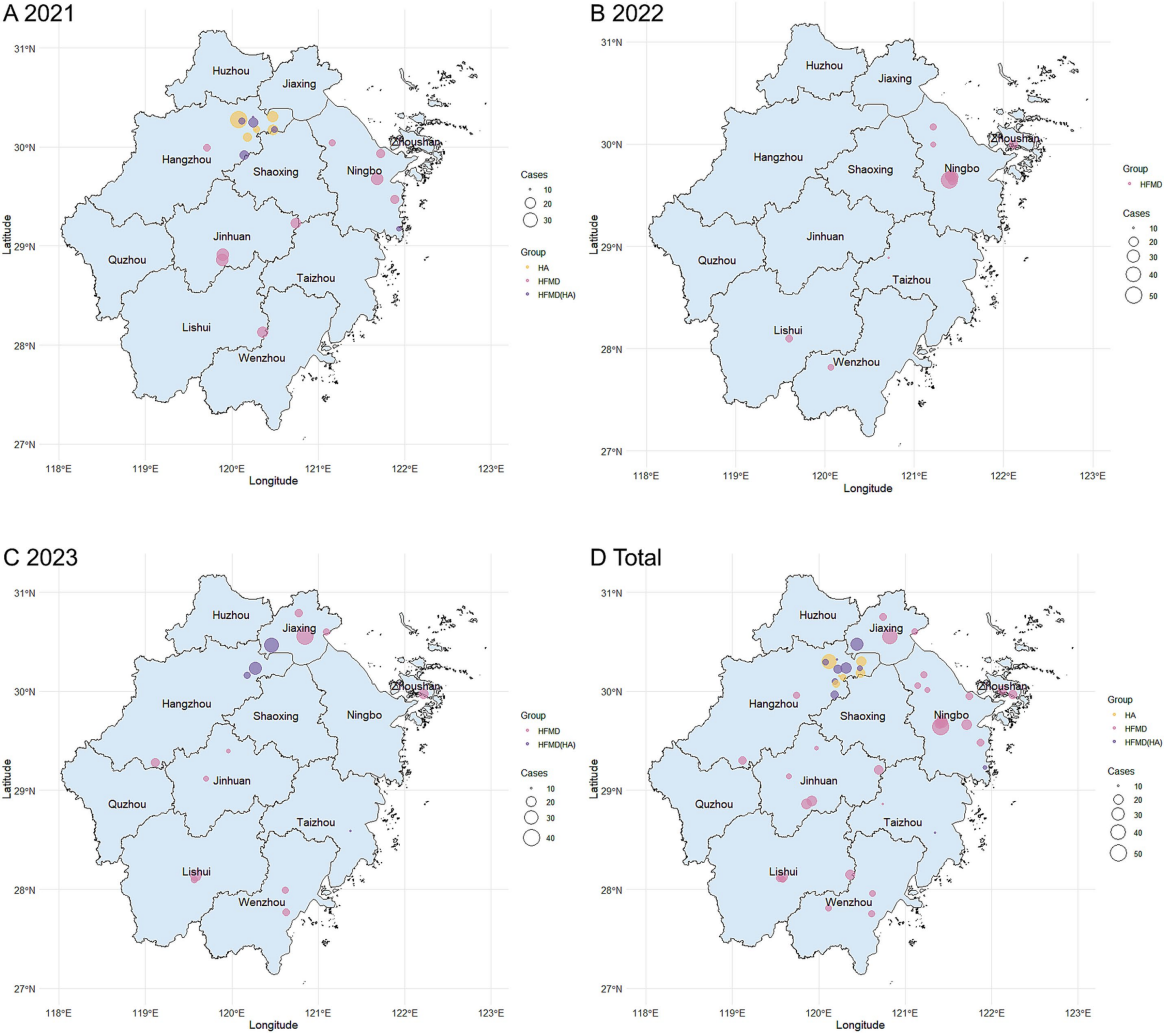
Geographical distribution of HFMD/HA outbreaks in Zhejiang Province, 2021–2023. **(A–C)** Illustrate the annual distribution of outbreaks for the years 2021, 2022, and 2023, respectively. **(D)** Shows the overall distribution of outbreaks from 2021 to 2023. The maps were created using R software (version 4.3.1; https://www.r-project.org/). Each dot on the map represents the exact location of an outbreak, with the size reflecting the scale of the outbreak. Yellow dots indicate HA outbreaks, red dots denote HFMD outbreaks, and purple dots represent mixed HFMD(HA) outbreaks.

In total, 47 confirmed outbreaks were reported across 28 counties in 8 cities within Zhejiang province. In 2021, all six HA outbreaks and five of the six HFMD(HA) outbreaks occurred in Hangzhou, including one major HA outbreak. Another HFMD(HA) outbreak occurred in Xiangshan County, Ningbo. Additionally, nine HFMD outbreaks were documented across four cities and eight counties, including four in Ningbo and three in Jinhua. In 2022, the majority of HFMD outbreaks (seven) were reported in Ningbo, including one major outbreak. In 2023, 11 HFMD outbreaks were recorded across six cities and 10 counties, along with four HFMD(HA) outbreaks in three cities and three counties. Notably, Jiaxing experienced four outbreaks in 2023, including two major outbreaks (Figure 2).

### Temporal distribution

**Figure 3:**
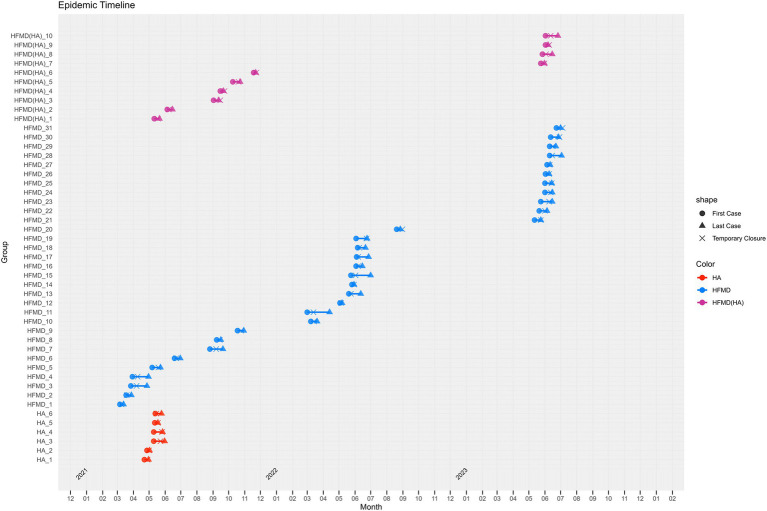
Time Distribution of HFMD/HA Outbreaks in Zhejiang Province, 2021–2023. Note: Each line in the figure represents an outbreak, with its length indicating the duration of the outbreak. Circles (●) denote the onset time of the first case, triangles (▲) represent the onset time of the last case, and crosses (❌) indicate the time of school closure. Red lines indicate HA outbreaks, blue lines denote HFMD outbreaks, and purple lines represent mixed HFMD(HA) outbreaks.

In 2021, outbreaks were observed from April to November, with HA outbreaks primarily occurring in April and May, while HFMD outbreaks were reported from March to October. HFMD(HA) outbreaks were recorded between May and November. In 2022, HFMD outbreaks were documented from March to August. In 2023, both HFMD and HFMD(HA) outbreaks were reported in May and June (Figure 3).

### Clinical characteristics

Herpangina outbreaks are associated with significantly higher proportions of fever (*p* < 0.01), oral vesicles (*p* < 0.01), and sore throat (*p* = 0.03) compared to HFMD outbreaks. Similarly, when comparing HFMD(HA) to HFMD outbreaks, HFMD(HA) exhibits a higher proportion of fever(*p* < 0.01) but a lower proportion of limb rash (*p* < 0.01). Furthermore, HA outbreaks show higher proportion of fever (*p* = 0.039) and oral vesicles (*p* < 0.01) compared to HFMD(HA) outbreaks (Table 1).

**Table 1:** Comparison of clinical characteristics of HFMD/HA cases in Zhejiang Province from 2021 to 2023.

	HFMD	HFMD(HA)	HA	χ^2^_1_	*P*-value	χ^2^_2_	*P*-value	χ^2^_3_	*P*-value
Gender(Male/Female)^b^	1.49:1(339/228)	1.63:1(98/60)	1.35:1(66/49)	0.139	1.000	0.173	1.000	0.418	1.000
Age	2–10 years old	3–9 years old	3–6 years old						
Clinical manifestations (percentage)	*N* = 338*	*N* = 117	*N* = 115						
Fever^b^	36.39%	75.21%	88.70%	91.823	<0.01	51.128	<0.01	6.230	0.039
Oral herpes^b^	79.88%	87.18%	100%	25.668	<0.01	2.634	0.315	13.714	<0.01
Sore throat^#,b^	25% (18/72)	30% (3/10)	51.28% (20/39)	6.638	0.03		1.000^a^		0.897^a^
Erythema on limbs	89.94%	54.70%		/	/	67.534	<0.01	/	/

(1) χ^2^_1_ compares HFMD with HA; χ^2^_2_ compares HFMD with HFMD(HA); χ^2^_3_ compares HA with HFMD(HA). (2) An asterisk (*) indicates that three HFMD outbreaks were investigated via telephone, resulting in 35 individuals with missing clinical data due to unreachable respondents; therefore, the denominator for these calculations is 338. (3) A hash sign (#) indicates that, in most outbreak investigations, sore throat symptoms were not given attention, and therefore the denominator was adjusted to the number of cases in outbreaks with descriptions of sore throat symptoms. (4) The letter “^a^” denotes the use of Fisher’s exact probability method for calculations. (5) The letter “^b^” signifies the use of the Bonferroni correction for *p*-values.

### Etiological results

**Figure 4:**
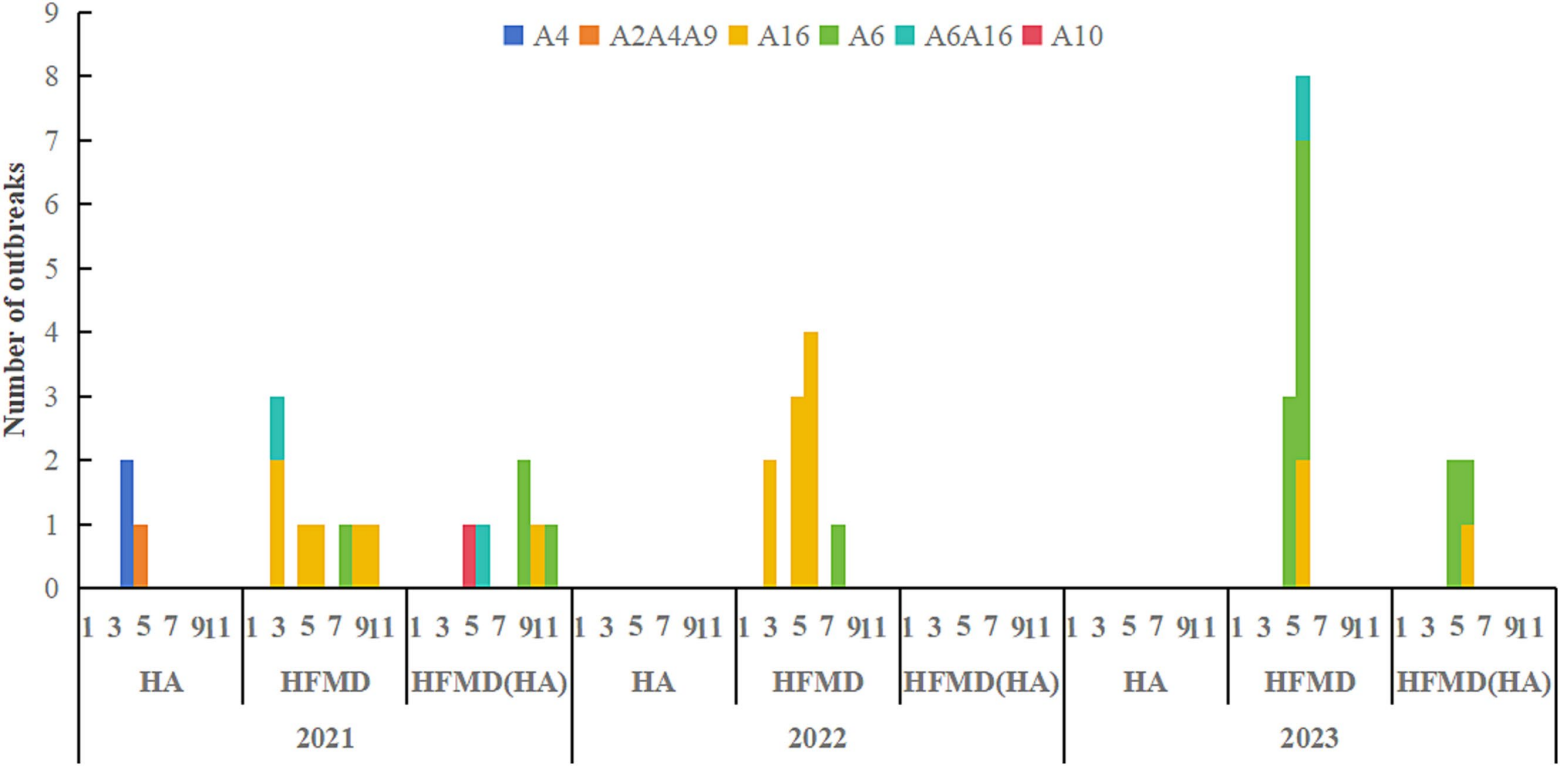
Composition of pathogens in different types of HFMD/HA outbreaks. A6A10 and A2A4A9 indicate the presence of multiple pathogens during one outbreak.

The reported HA outbreaks involved in total of 115 cases, with pathogen tests conducted on 62 patients. The enterovirus positivity rate was 67.74% (42/62), with the specific pathogen type was identified in 22 cases (22/42, 52.38%). In one outbreak, an asymptomatic preschool teacher from the affected class tested positive for CVA. The HMFD outbreaks involved 567 cases. Pathogen testing on 254 samples revealed an enterovirus positivity rate of 82.28% (209/254), with specific pathogen types identified in 175 cases (175/209, 83.73%). Among four outbreaks caused by CVA16, the positivity rate among close contacts was 37.04% (10/27). Additionally, the mixed HMFD (HA) outbreaks involved 158 cases. Pathogen testing on 93 patients showed an enterovirus positivity rate of 68.82% (64/93), with specific pathogens identified in 55 cases (55/64, 85.94%). In one outbreak caused by CVA6, the positive detection rate among close contacts was 22.22% (6/27), including three family members (father, mother, and sister) of one affected child. Details of the specific pathogen composition are presented in Figure 4 and Table 2.

**Table 2:** Distribution of pathogens in laboratory diagnoses of various diseases.

Disease classification	Year	Sample	Laboratory diagnosis	CVA2	CVA4	CVA6	CVA9	CVA10	CVA16	Mixed positive	Non-specific positive but not subtyped
HA	2021	62	42	1(2.38%)	18(42.86%)		1(2.38%)			2(4.76%) (A4A9)	20(47.62%)
HFMD	2021	107	88			14(15.91%)			56(63.64%)		18(20.45%)
	2022	58	58			3(5.17%)			47(81.03%)		8(13.79%)
	2023	89	63			43(68.25%)			11(17.46%)	1(1.59%) (A6A16)	8(12.70%)
HFMD(HA)	2021	37	33			17(51.52%)		1(3.03%)	5(15.15%)	1(3.03%) (A6A16)	9(27.27%)
	2023	56	31			17(54.84%)			14(45.16%)		

The percentage in parentheses is calculated as follows: number of positive cases/number of laboratory diagnosed cases * 100%.

### Control timeliness assessment

The duration of an outbreak shows a statistically significant positive correlation with the number of cases (coefficient = 0.064, *p* < 0.001), indicating that each additional day of outbreak duration results in an approximate 6.6% increase in case numbers [exp(0.064) = 1.066]. Furthermore, the timing of class suspension has a statistically significant impact (*p* = 0.022); each day’s delay in initiating class suspension is associated with an approximate 5.0% increase in case numbers [exp(0.048) = 1.050]. The interaction coefficient between the timing of class suspension and the duration of the pandemic is −0.004, indicating a small but statistically significant negative association (*p* = 0.003). In the model, the coefficients for HFMD (coef = −0.259; *p* = 0.016) and HFMD(HA) (coef = −0.300; *p* = 0.021) are statistically significant. This suggests that, after adjusting for other variables in the model, the presence of HFMD or HFMD(HA) outbreaks is associated with a decrease in the expected number of HA outbreaks. The model, with an adjusted R^2^ of 81.0%, indicates that it accounts for a substantial proportion of the variability in HA outbreak cases (Table 3).

**Table 3:** Evaluation results of the GLM based on Poisson distribution.

	Coef	Std err	*Z*-value	*P-*value	OR	95%CI
Const	2.235	0.188	11.891	<0.001	9.342	6.434–13.444
HFMD	−0.259	0.107	−2.407	0.016	0.772	0.626–0.957
HFMD(HA)	−0.300	0.130	−2.301	0.021	0.741	0.574–0.957
Duration	0.064	0.012	5.362	<0.001	1.066	1.041–1.091
start_to_suspend	0.048	0.021	2.288	0.022	1.050	1.007–1.094
duration:start_to_suspend	−0.004	0.001	−2.987	0.003	0.996	0.994–0.999

## Discussion

Hand, foot, and mouth disease and herpangina are highly contagious pediatric infections caused by enteroviruses, capable of spreading through multiple routes and causing widespread outbreaks. In Zhejiang Province, with a population of approximately 65 million (4.6% of China’s total)—the HFMD surveillance system covers all 90 counties and districts, enabling comprehensive data collection from diverse healthcare settings. Nationally, data from the China CDC indicate that China recorded 1,354,548 HFMD cases in 2021, including eight fatalities (27). Of these, Zhejiang Province reported 123,967 cases of HFMD in 2021, including one fatality. Among these cases, our study focused on approximately 0.2%, specifically those associated with outbreaks in group settings. In China, kindergartens typically serve children aged 3–6 years, while elementary schools cater to those aged 6–12 years. The majority of HFMD cases are sporadic, occurring in children under the age of three who are not enrolled in group settings. Between 2021 and 2023, Zhejiang Province reported 49 outbreaks of HFMD and HA, all of which occurred in schools and childcare facilities, with 89.80% occurring in childcare facilities, including kindergartens and nurseries. Notably, 11 outbreaks involving HA or mixed HA/HFMD were exclusively reported in Hangzhou. This may be related to the fact that HA is not classified as a legally notifiable disease in China, and the absence of a systematic surveillance platform has hindered accurate assessment of its true prevalence and outbreak characteristics. However, the high sensitivity and vigilance of Hangzhou’s surveillance system in detecting HA outbreaks likely contributed to this reporting pattern.

A variety of enteroviruses, including EV71, Coxsackievirus, and Echovirus, are responsible for causing HA and HFMD, with overlapping pathogen spectra prevalent in both diseases. Surveillance of sporadic HA and HFMD cases in Jiangsu Province from 2013 to 2014 identified EV71, CVA16, CVA24, and CVA10 as the dominant pathogens (12). While some studies have reported significant differences in the predominant viral strains between HA and HFMD (28). Our study found that HA outbreaks were predominantly caused by CVA4, along with other enteroviruses such as CVA2 and CVA9. In contrast, HFMD outbreaks were predominantly associated with CVA6 and CVA16, which were also frequently linked to oral herpes cases. Although HA and HFMD share similarities in their epidemiological characteristics—both primarily affecting young children in childcare facilities and peaking in the spring and summer—HFMD demonstrates a broader temporal distribution. This difference may be attributed to variations in the dominant serotypes between the two diseases. In 2021, CVA4 was identified as the dominant serotype in HA outbreaks in Hangzhou (29), contrasting with the predominance of CVA2 in sporadic HA cases in 2015 but aligning with the CVA4 dominance observed in sporadic cases in 2016 (11). The epidemiological differences between HA and HFMD are closely linked to variations in their dominant serotypes.

Clinically, HA is characterized by the sudden onset of fever accompanied by vesicles or ulcers in the oralpharynx, typically without an associated skin rash. This condition is often observed in infants who experience difficulty eating due to oral pain. There is potential for clinical overlap between HA and HFMD. Liu et al. (30) reported a yearly decline in the incidence of hand and foot rashes among severe HFMD cases in Shenzhen from 2008 to 2012, alongside a significant increase in oral vesicles. In contrast, a study by Yao et al. (12) comparing HA and HFMD found that fever was more frequently observed in HA cases, with higher average temperatures, suggesting greater neurological involvement and a potential for more severe disease progression. Our study further reveals that fever and sore throat are more commonly observed in HA than in HFMD. Close monitoring is warranted for HA cases with persistent high fever, as they may be at an increased risk of developing severe complications.

Children’s caregivers with latent enterovirus infection might become a potential source of HFMD/HA transmission among children (31). This study included the detection of asymptomatic infections among close contacts of HFMD and HA cases, including adults, in certain outbreak events. The positive detection rates for close contacts during outbreaks caused by CVA16 and CVA6 were 37.04 and 22.22%, respectively. Research on the transmission of enteroviruses suggests that class or school closures may help contain the spread of outbreaks (31), although the supporting evidence remains limited (32).

Using a Poisson GLM, this study identifies outbreak duration as a critical factor influencing outbreak scale. Specifically, each additional day of an outbreak is associated with an approximate 6.6% increase in case numbers. These findings underscore the importance of timely interventions to control outbreaks spread. Implementing control measures, such as school closures, shortly after an outbreak begins is essential. Early detection and prompt action can significantly reduce both the duration and impact of the outbreak, emphasizing the need for rapid response strategies in epidemic management.

Several limitations of this study must be taken into account. Firstly, the data analyzed primarily originate from survey reports within the information management system. As HA is not a legally notifiable infectious disease, regional variability in surveillance and early warning systems likely contributes to underreporting. Although the COVID-19 pandemic improved surveillance sensitivity for HA, its non-notifiable status continues to limit accurate reporting. Secondly, this study focuses exclusively on the epidemiological characteristics and intervention practices of HFMD and HA outbreaks in Zhejiang Province, which may not fully reflect the epidemic features and current situation across mainland China.

In conclusion, this study provides the first comprehensive analysis of outbreak characteristics and the timeliness of interventions for enterovirus-related diseases across Zhejiang Province, China. Outbreaks of HFMD and HA are caused by different enteroviruses, with CVA6 and CVA16 being the predominant pathogens in HFMD, and CVA4 in HA. Childcare facilities are critical settings for these outbreaks, where prompt isolation and effective management of infected individuals are critical measures for preventing and controlling the spread of these diseases.

## Data Availability

The raw data supporting the conclusions of this article will be made available by the authors, without undue reservation.
